# Protocol for expansion of an existing national monthly survey of smoking behaviour and alcohol use in England to Scotland and Wales: The Smoking and Alcohol Toolkit Study

**DOI:** 10.12688/wellcomeopenres.16700.1

**Published:** 2021-03-29

**Authors:** Loren Kock, Lion Shahab, Graham Moore, Emma Beard, Linda Bauld, Garth Reid, Leonie Brose, Marie Horton, Ashley Gould, Jamie Brown

**Affiliations:** 1 Behavioural Science and Health, University College London, London, UK; 2 DECIPHer, School of Social Sciences, Cardiff University, Cardiff, UK; 3 Usher Institute, College of Medicine and Veterinary Medicine, University of Edinburgh, Edinburgh, UK; 4 Public Health Scotland, Edinburgh, UK; 5 Institute of Psychiatry, Psychology and Neuroscience, Kings College London, London, UK; 6 Population Health Analysis, Health Intelligence, Public Health England, London, UK; 7 Public Health Wales, Cardiff, UK

**Keywords:** Survey, public health, policy, health behaviour, tobacco, alcohol

## Abstract

Background

The Smoking and Alcohol Toolkit Study (STS/ATS) in England has delivered timely insights to inform and evaluate strategies aimed at reducing tobacco smoking- and alcohol-related harm. From the end of 2020 until at least 2024 the STS/ATS is expanding to Scotland and Wales to include all constituent nations in Great Britain. Expanding data collection to Scotland and Wales will permit the evaluation of how smoking and alcohol related behaviours respond to divergent policy scenarios across the devolved nations.

Methods

The STS/ATS consists of monthly cross-sectional household interviews (computer or telephone assisted) of representative samples of adults in Great Britain aged 16+ years. Commencing in October 2020 each month a new sample of approximately 1700 adults in England, 450 adults in Scotland and 300 adults in Wales complete the survey (~n = 29,400 per year). The expansion of the survey to Scotland and Wales has been funded for the collection of at least 48 waves of data across four years. The data collected cover a broad range of smoking and alcohol-related parameters (including but not limited to smoking status, cigarette/nicotine dependence, route to quit smoking, prevalence and frequency of hazardous drinking, attempts and motivation to reduce alcohol consumption, help sought and motives for attempts to reduce alcohol intake) and socio-demographic characteristics (including but not limited to age, gender, region, socio-economic position) and will be reviewed monthly and refined in response to evolving policy needs and public interests. All data analyses will be pre-specified and available on a free online platform. A dedicated website will publish descriptive data on important trends each month.

Discussion

The Smoking and Alcohol Toolkit Study will provide timely monitoring of smoking and alcohol related behaviours to inform and evaluate national policies across Great Britain.

## Introduction

Each year in the devolved nations of Great Britain (England, Scotland and Wales) tobacco smoking and alcohol use together are estimated to cause over 90,000 deaths and more than 1 million hospital admissions ^1– 3^. The level of tobacco smoking has declined over the past decade, but remains prevalent with between one in five and one in seven adults smoking across all devolved nations ^2^. Over a similar time period the consumption of alcohol and the number of people reporting drinking has also decreased, but less dramatically ^4^. However, reductions in both behaviours and the harms associated with them have not been uniform across the population with inequalities persisting according to certain characteristics such as socio-economic position, mental health status, sex and age ^1, 2, 5^.

Representative population surveys are an important resource to inform and evaluate key GB policies on tobacco and alcohol use. The Smoking and Alcohol Toolkit Study (STS/ATS) has been collecting detailed data in England on smoking behaviour since November 2006, and alcohol use behaviour since March 2014. The STS/ATS reports monthly on key smoking ^6^ and alcohol ^7^ indicators (and their interdependencies ^8^) and has the granularity to detect trends and socio-demographic differences ^5^ that would otherwise be missed by surveys that collect data less frequently ( Table 1). This enables researchers using the STS/ATS to have greater confidence when assessing whether important events and policies have led to changes in the prevalence and trends in smoking and alcohol use behaviours. For instance, STS/ATS data from England has enabled evaluation of the impact of specific population-level policies - such as expenditure on tobacco control mass media campaigns, and changes in national drinking guidelines - on smoking and alcohol use behaviour ^6, 9^.

**Table 1.** Characteristics of current representative surveys that collect data on tobacco and alcohol use among adults.

Representative Survey	Behaviour measured (tobacco/alcohol/ tobacco and alcohol)	Sample	Design	Tobacco/alcohol control parameters measured
STS/ATS	Smoking and alcohol	Data collected on ~34,200 adults in GB each year aged 16+.	Monthly cross-sectional surveys with 6 months follow-up.	Smoking status; amount smoked and nicotine intake; nicotine dependence; route to quit; motivation to smoke; harm reduction; e-cigarette use; support for policy measures
				Current attempts to cut down consumption; GP/health-care professional advice; type of alcohol consumed; motivation to cut down and consumption; amount spent; strength of urges to drink; number of recent serious attempts to cut down; aids used to help cut down; motives for attempts to cut down
HSE ^12^	Smoking and alcohol	Data collected on 5000–15000 adults each year (aged 16+). Since 1995, it has also included children aged 2–15 years of age. Information on children under 13 is obtained from a parent.	Survey conducted annually.	Smoking prevalence; cigarette consumption; cigarette type; cigarette dependence; salivary cotinine (biochemical indicator of cigarette smoke intake); e-cigarette use; intention to quit smoking; demographics
				Frequency of drinking in the last 12 months; number of days drank previous week; on the heaviest drinking day, the types and amount of alcoholic drinks consumed; for those who drank in the last 12 months, the frequency of drinking different types of drink and the amounts of each drunk on a typical day.
UKHLS ^13^	Smoking and alcohol	Data collected on 40,000 households. Adult household members aged 16 or older are given the full-length questionnaire and those aged 10–15 years of age are asked to complete a shorter version.	Panel survey of households with yearly interviews. Data collection for a single wave is scheduled across 24 months.	Smoking status; amount smoked and nicotine intake; nicotine dependence; route to quit; e-cigarette use;
				Past month alcohol spend; frequency of alcohol consumption whilst pregnant; units of alcohol per day/week; ever consumption of alcohol; number of alcoholic drinks in past month
OPN ^14^	Smoking	Data collected on ~8,000 adults per year (aged 16+).	Monthly survey. Surveys runs for eight months each year.	Smoking prevalence; cigarette consumption; cigarette type; cigarette dependence; tar yield; age started smoking; desire to quit; e-cigarette and other alternative nicotine delivery device use demographics.
APS (Labour Force Survey) ^15^	Smoking	Household surveys across the UK. N = ~320,000	Survey conducted monthly (disseminated quarterly and reported annually).	Regular and current cigarette smoking.
ITC ^16^	Smoking	20 countries (including UK); telephone surveys. N = ~2,000 adult smokers per country.	Annual. Yearly follow-up with replenishment for drop-outs and those who stop smoking.	Smoking behaviours and dependence; quitting behaviours; use of alternative nicotine products; attitudes towards and effects of label warnings, advertising, and taxation; health beliefs; demographics; other potential moderators.
YouGov/ASH Smokefree GB	Smoking	2010 to present, N = ~12,000, 18+ GB population	Cross-sectional surveys conducted annually in Feb/March each year	Smoking status, e-cigarette use, cannabis use (2019 onwards) smokeless tobacco, shisha and other nicotine containing products (not in all years). Exposure to second- hand smoke and support for policy measures.
National survey for Wales ^17^	Smoking & Alcohol	~12,000 adults across Wales	Cross-sectional annual and monthly surveys	Smoking status and quitting behaviour, e-cigarette use, exposure to second-hand smoke
				Alcohol consumption
Scottish health survey ^18^	Smoking & Alcohol	~4800 adults age 16+ across Scotland	Annual cross-sectional surveys	Smoking status and quitting behaviour, cigarette consumption, exposure to second-hand smoke, NRT use, e-cigarette use, medical advice on smoking cessation
				Alcohol consumption, type of alcohol consumed
NDNS ^19^	Alcohol	2008 to present surveys cover all individuals’ aged 1.5 years+ living in private households. Data are currently collected on around 500 adults (aged 19+) and 500 children each year (aged 1.5–18 years).	Cross-sectional surveys conducted annually.	Alcohol consumption in the past week; on the heaviest drinking day, amount drunk; type of alcohol consumed
IAC APISE ^20^	Alcohol	~2000 adults (aged 16+) in England.	Annual survey with 12 month follow-up.	Frequency of drinking and typical occasion quantity; preloading; drinking location (e.g. pub, own home); perceptions of availability and affordability

STS/ATS: Smoking and Alcohol Toolkit Study; HSE = Health Survey for England; ONS = Office of National Statistics Opinions Survey (formerly Omnibus Survey); UKHLS = Understanding Society, the UK Household Longitudinal Study; APS = Annual Population Survey; OPN = Opinions and Lifestyle Survey; ITC = International Tobacco Control Policy Evaluation Project; NDNS = National Diet and Nutrition Survey; IAC APISE = International Alcohol Control Policy Evaluation Study: England and Scotland study.

The expansion of the STS/ATS data collection into Scotland and Wales will provide new insights into smoking and drinking behaviours within each nation and enable the comparison of trends across Great Britain in response to divergent devolved government policies.

The purpose of this paper is to provide an overview of the STS/ATS methodology and detail any specific changes that are relevant following the inclusion of new data collection in Scotland and Wales, and the effect of the coronavirus disease 2019 (COVID-19) pandemic.

## Aims

The STS/ATS aims to provide regular tracking of smoking and alcohol-related behaviours on a monthly basis to inform and evaluate national tobacco and alcohol control policies across devolved nations in Great Britain. It will also allow analyses to be conducted as a function of important socio-demographic variables. Monthly and quarterly trends will be published on the variables listed below. Overall prevalence will be presented quarterly by age group, social grade, region and gender, while the socio-demographic stratification will be reported annually for all other outcomes.

### Smoking related outcomes

1. Cigarette smoking prevalence
2. Smoking and quitting behaviour (number and success rate of quit attempts, motivation to quit, cigarette dependence)
3. Support/method used to quit smoking
4. Prevalence of electronic cigarette (e-cigarette/vape) use

### Alcohol related outcomes

1. Prevalence of hazardous drinking (measured using the Alcohol Use Disorders Identification Test (AUDIT))
2. Hazardous drinkers who report attempting to reduce their alcohol consumption
3. Methods used by hazardous drinkers attempting to reduce their consumption
4. Hazardous drinkers who report receipt of advice to reduce alcohol consumption from a health professional in the past year

Changes in these variables associated with events and policies, such as the introduction of new product pricing or sales and restrictions, will also be evaluated.

## Methods

### Design

The STS/ATS involves monthly cross-sectional household computer-assisted interviews, conducted by Ipsos Mori. Each month a sample of approximately 2,450 adults aged 16+ years and over in Great Britain (1,700 in England, 450 in Scotland, 300 in Wales) is included. Data for the STS in England were first collected in November 2006. Data for the ATS in England were first collected in March 2014. The expansion of STS/ATS data collection to include Scotland and Wales commenced in October 2020.

Sampling methods for the STS/ATS have been described previously ^10, 11^. Briefly, the survey uses a form of random location sampling. Great Britain is first split into 227,403 ‘Output Areas’, each comprising of approximately 300 households. These areas are then stratified according to established geo-demographic characteristics and geographic region then randomly selected into an interviewer’s list.

Before the COVID-19 crisis, these areas were randomly allocated to interviewers, who travel to their selected areas to conduct electronic interviews with one member of the household aged 16 years or over. Interviews are conducted until quotas based upon factors influencing the probability of being at home (i.e. working status, age and gender) are fulfilled. Morning interviews are avoided to maximise participant availability. However, due to social distancing measures to address COVID-19, in-person interviews were halted in March 2020 and replaced temporarily with telephone interviews among adults aged 18 years or older until such time that is safe to return to the usual method.

The telephone interviews are conducted by landline and mobile using a standard landline random digit dialling (RDD), mobile RDD, and targeted mobile. In terms of the sample processed, each eligible landline telephone number across GB has a random probability of selection proportionate to population distribution (i.e. stratification of the landline telephone database by and within Government Office Region, GOR). Within GOR, the system is based on UK postcode sector information. Each postcode sector is matched to the relevant standard telephone dialling code and telephone number stubs are derived from information obtained from the Office of Communications (Ofcom). Selection probability of postcode sectors is proportional to the number of households within or across a given area by using the household density information that is attached to each postcode sector. Mobile sampling uses largely the same approach as landline sampling; however, the selection is in proportion to the known mobile network share. This mobile network share is continually updated using robust publicly available statistics to ensure that accurate samples of the mobile using population. Mobile, targeted mobile and landline sampling are carried out in equal proportions. To maximise response rates more landline sampling takes places earlier in the day, with more mobile sampling later in the day.

Data are provided to UCL in a SPSS dataset, with analyses typically undertaken in the latest version of R or SPSS.

### Ethical considerations

In accordance with our ethical approval (ID 2808/005), all respondents are given a written information sheet about the study and provide informed verbal consent. Ipsos MORI handle all personal data according to GDPR. Ethical approval for the STS/ATS and expansion to Scotland and Wales was granted by the UCL Ethics Committee as was the change to telephone sampling (ID 0498/001).

### Sample

The study is initially funded for a four-year period between October 2020 and September 2024. It is anticipated that data on around 29,400 individuals (~20,400 from England, 5,400 from Scotland and 3,600 from Wales) will be collected each year. However, as with the STS and ATS which have been collecting data in England for 14 and 6 years, respectively, it is planned that the STS/ATS will be extended beyond this initial period in Wales and Scotland.

### Measures

A core set of measures are included in each monthly STS/ATS survey. The STS/ATS was set up as a dynamic ‘toolkit’ for researchers in alcohol and tobacco control, and has the flexibility to add or remove questions as needed to test new hypotheses or according to policymaker and public interest across GB. For instance, the STS/ATS questionnaires were revised in March 2020 following consultation with a range of stakeholders across Great Britain and a selection of new questions and response options were added.

The measures in the STS/ATS are summarised in Table 2 and below. Specific details regarding the questions and responses for each measure are provided as extended data ^21^. The majority of these measures have also been reported in previous protocols for the smoking and alcohol components of the survey ^10, 11^.

**Table 2.** Summary of STS/ATS measures.

Measures	
Sociodemographic characteristics	Sex
	Ethnicity
	Socio-economic position (NRS occupation based social grade ^22^, housing tenure, car ownership, educational achievement, income, employment status)
	Age
	Government office region
	Marital status
	Number of adults and children in household
	Disability
	Religion
	Internet access and use
	Newspaper readership
	Electronic durables ownership and use
Smoking measures	Smoking status
	Amount smoked and nicotine intake
	Nicotine dependence
	Route to quit smoking
	Motivation to stop smoking
	Harm reduction
	Source of tobacco purchase
	Menthol cigarette consumption ^a^
	Support for current policy options ^b^
Alcohol measures	Prevalence & frequency of hazardous drinking
	Current attempts and motivation to reduce alcohol consumption
	Receipt of health professional advice on drinking
	Amount spent
	Urges to drink
	Serious attempt to reduce intake
	Help sought and motives for recent attempts to reduce intake
	Source of alcohol purchase ^c^
	Exposure to drinking guidelines ^c^
	Support for alcohol policy options ^b^
Mental health measures	Diagnosis with a mental health problem (since aged 16 years) ^d^
	Past month psychological distress ^d^

^a^Added in July 2020 following EU TPD ban on sale menthol cigarettes; ^b^Asked for one wave (November) annually; ^c^Added following consultation with stakeholders; ^d^Mental health question added in April 2020

***Sociodemographic characteristics.*** Data are collected on sex, ethnicity, socio-economic position (SEP), marital status, number of residents and children in the household, sexual orientation, age, disability, religion, internet access and use, newspaper readership, durable electronic goods ownership, and government office region (London, South East, South West, East Anglia, East Midlands, West Midlands, Yorkshire/Humberside, North West and North East). Various measures are used as indicators of SEP, such as car and home ownership, employment status, educational achievements, income, housing tenure and by social grade. Social grade is measured using the National Readership Survey social-grades system: A: higher managerial, administrative or professional; B: intermediate managerial, administrative or professional; C1: supervisory or clerical and junior managerial administrative or professional; C2: skilled manual workers; D: Semi and unskilled manual workers; and E: Causal workers, pensioners and others who depend on the welfare state for their income ^22, 23^.

***Smoking measures***

**Smoking status**

Respondents provide information as to whether they are currently smoking (daily or non-daily), quit within the past year, quit more than one year ago, never smoked, or if they use non-cigarette tobacco (e.g. cigar/pipe smoking).

**Amount smoked and nicotine intake**

To ascertain consumption, participants are asked about the number of cigarettes or other nicotine products used per day, week or month.

**Nicotine dependence**

To assess nicotine dependence, respondents answer questions used to derive a value for the Fagerström Test for Nicotine Dependence, the Heaviness of Smoking Index and the strength of urge to smoke ^24, 25^.

**Route to quit**

Respondents are asked questions pertaining to their motivation to quit smoking, the triggers of quit attempts, barriers to quitting, attempts to quit, methods of quitting (including pharmacological and behavioural aids, health professional advice, planning in advance, pre-quit smoking reduction) and success at quitting.

**Motivation to stop smoking**

To provide information on motivation to stop smoking, participants are asked about their attitudes, beliefs and motives associated with smoking and smoking cessation ^26^.

**Harm reduction**

Harm reduction measures include questions about attempts to cut down but not quit; use of nicotine replacement therapy for cutting down and/or temporary abstinence from smoking, and the use of e-cigarettes.

**Support for current policy options**

For one wave annually (May) participants are asked about their level of support for various policies (e.g. raising the legal age of tobacco sale, tax increases, cigarette pack inserts).

***Policy specific items.*** Questions can be added or taken away in response to relevant ongoing and emerging policy issues. An example of this is below with regard to menthol cigarettes.

**Menthol cigarettes**

Under the European Union Tobacco Products Directive, from May 20 ^th^ 2020 the sale of menthol cigarettes was banned in the UK ^27^. To understand whether this ban has impacted menthol cigarette smoking an additional STS/ATS question was added in July 2020 and respondents are now asked about the flavour of tobacco they usually smoke. 

***Alcohol measures***

**Prevalence and frequency of hazardous drinking**

The AUDIT screening tool ^28^ for alcohol use is used to identify participants who could be classed as dependent, harmful or hazardous drinkers. The STS/ATS uses an extended item version of the AUDIT questionnaire adopted in previous ‘Screening and Brief Alcohol Intervention in Primary Care (SIPS)’ trials; to allow exploration of higher levels of alcohol consumption in addition to the standard AUDIT scoring system ^28^. Those who indicate hazardous and or harmful alcohol consumption and possible dependence are then asked a set of questions summarised below.

**Current attempts and motivation to reduce alcohol consumption**

Participants are asked if they are currently attempting to reduce their alcohol intake and whether they are motivated to do so.

**Receipt of health professional advice on drinking**

Participants are asked if they received any advice about their alcohol consumption from their GP and the form of this advice i.e. whether they were given help during a GP surgery visit or referred to a specialist service.

**Amount spent**

Participants provide information on the amount they spend on alcohol per week, and how much of this is spent buying alcohol on premises away from their home (e.g. pubs, clubs, bars or restaurants).

**Urges to drink**

Participants are asked how strongly they have felt the urge to drink alcohol in the past 24 hours.

**Serious attempt to reduce intake**

The number of serious attempts to reduce alcohol intake is reported by participants.

**Help sought and reasons for recent attempts to reduce intake**

Related to the recent attempts to cut down on drinking, questions are also asked on what help was sought (e.g. medication, counselling, a website or telephone helpline, low-alcohol/alcohol free drinks) and what the reasons were for cutting down (e.g. advice from doctors, expense, advertisements or health problems).

**Source of alcohol purchase**

Participants provide information about where they purchase alcohol (for their own consumption).

**Exposure to drinking guidelines**

Participants are asked about where they saw, read or heard about national drinking guidelines.

**Support for current policy options**

As with the tobacco policy questions, for one wave annually (May) participants are asked about their level of support for various policies (e.g. minimum unit pricing, sales restrictions, taxes).

***Mental health measures.*** In 2016 and 2017 the STS/ATS asked past-year smokers in England about their mental health, asking about whether they had ever been diagnosed with any of a list of mental health problems, whether they had received treatment in the past year and their level of past-month distress using the K6, a screening instrument for severe and moderate mental illness ^29^). Two of these questions (ever diagnosis and past-month distress) have been included again from April 2020 and are now asked of **all** adults in GB. Due to the COVID-19 pandemic mental health questions are asked over the telephone, but when the face-to-face survey resumes they will be self-completed by each participant.

### Data management

Data from the STS/ATS is anonymous and does not contain any information that can be used to identify participants. Each month a new wave of cross-sectional data is sent by Ipsos Mori to UCL, where it is screened for error by UCL researchers before being cumulatively added to the previous wave(s). The anonymous dataset is shared with approved researchers at UCL and partner institutions for analysis. All procedures are GDPR compliant. New requests for access to data are approved following submission of a form detailing the purpose of the request and plans for analysis.

### Statistical analysis

Descriptive statistics (including parameter estimates, number of participants, measures of spread) will describe fundamental features overall and from each nation in GB, including smoking and alcohol related behaviours and the sociodemographic profile of participants.

For the reporting of individual-level descriptive statistics and for analyses involving data aggregated at the population-level, data is weighted to be representative of the population using a rim (marginal) weighting technique ^30^. This involves an iterative sequence of weighting adjustments whereby separate nationally representative target profiles are set (gender, working status, prevalence of children in the household, age, social grade and region) and the process repeated until all variables match the specified targets.

Although causality cannot conﬁdently be inferred from observational designs, analyses using STS/ATS data will measure and adjust for potential confounding. At the individual level this may involve including socio-demographic characteristics; at the population level this will involve other interventions and policies that have been introduced in that population.

***Differences between groups.*** To assess group differences (e.g. according to socio-demographic or smoking and drinking characteristics of interest) where the outcome is normally distributed we will use independent samples t-tests (to compare the means of a quantitative dependent variable across two levels of an independent variable) and ANOVAS (for differences in the means of a quantitative dependent variable across two or more levels of an independent variable) or ANCOVAS (where there are continuous and categorical independent variables). Should parametric assumptions be violated, transformations will be performed or appropriate non-parametric tests employed (Wilcoxon-Mann-Whitney test and Kruskal Wallis test).

***Relationships between two or more variables.*** To describe the strength of a relationship between two categorical variables (for instance between certain characteristics of smoking and mental health status ^31^), chi-squared tests (or log-linear analysis for more than two categorical variables) will be used. We will use the generalized linear modelling (GLM) framework to examine the relationship between an independent and outcome variable. To examine the association between a quantitative independent variable and a normally distributed quantitative outcome variable we will use linear regression and multivariable linear regression (to adjust for confounding variables), as exemplified by an STS analysis assessing the number of cigarettes smoked per day by adult smokers from 2008 to 2017 ^6^.

For outcomes with other probability distributions (including binomial, Poisson and multinomial) we will use appropriate regression techniques from the GLM family. For binary outcome data we will use as binary and multivariable binary logistic (for odds ratios) or log-binomial (for relative risks) regression. These methods have been employed in numerous STS studies to analyse associations between self-reported abstinence and use of different smoking cessation aids ^32, 33^. Where the outcomes are ordinal or have more than two levels we will use ordinal or multinomial logistic regression, respectively. Ordinal regression was used to explore patterns of alcohol consumption in England throughout the year ^7^. For count data (e.g. the number of smokers per 1000 individuals each month ^34^) we will use Poisson or negative binomial regression models.

To assess the association between two or more independent variables with two or more dependent variables (for example between socio-economic position and several measures of armful alcohol drinking ^5^), multivariate regression will be used.

***Time-series analysis.*** To assess trends over time in response to policies and population-level interventions, interrupted time-series analysis (TSA) will be used. This family of analyses allows for autocorrelation and consideration of underlying trends in the time series ^35^. For data aggregated at the population level, methods of TSA include generalized least squares (GLS), generalized additive mixed models (GAMM), autoregressive integrated moving average with exogenous input (ARIMAX) and vector autoregression (VAR) models. Some of these methods have been applied to STS/ATS data to evaluate the effects of notable policies such as the revised low-risk drinking guidelines ^36^, and tobacco control mass media expenditure ^37^.

***Trend analysis.*** In the absence of autocorrelation, generalised linear models (including Poisson regression for low frequency data, and logistic regression for binary data) and linear regression (including polynomial regression, power and logarithmic regression) ^38^ will be used to assess an outcome across over a time trend. If the study is concerned with the impact of an event or intervention, segmented regression ^39^ will be used.

***Bayes factors.*** Bayes factors will be calculated where appropriate. They indicate the relative likelihood of a hypothesised difference/association versus no difference/association; and allow two interpretations of a null result to be distinguished (there is evidence for the null-hypothesis or the data are insensitive in distinguishing an effect) ^40^. Robustness regions may also be specified for reported Bayes factors, which indicate the range of expected effect sizes used when specifying the alternative model that support the same conclusion (e.g. evidence for hypothesised difference/association, evidence for the null hypothesis, or inconclusive outcomes) ^34^.

***Handling of missing data.*** Missing data on the outcome and other variables of interest will be handled using appropriate methods for each study and the type of missingness. First, we will assess whether respondents with missing data differ systematically from those with complete data on the exposure and outcome variables of interest. If data are missing from variables in a small number of cases (<5%), these cases will be removed from the analytical sample and a complete-case analysis will be conducted under the assumption that they are missing completely at random ^41^. In situations where data on outcome variables were not collected for every wave during a given time-period, multiple imputation will be used to impute the missing values (using all other variables as predictors (complete cases only)). For time-series analyses that are being conducted on aggregated data from surveys that have differing amounts and types of missing data in the samples generating the aggregate value, time-series specific multiple imputation methods will be used ^35^.

***Pre-registration.*** All analysis plans will be pre-registered on the open science framework prior to conducting the analysis. This includes considering and planning the inclusion of potential confounding variables.

### Dissemination

Results will be disseminated using a website with findings shown for each nation in Scotland, Wales and England. Regular updates will be delivered to key stakeholders across these nations including devolved governments, key public health bodies and non-governmental organisations working in tobacco or alcohol control.

## Discussion

Extension of the STS/ATS from England into Scotland and Wales will provide timely representative data on smoking and alcohol use across Great Britain, and permit monitoring of trends in behaviour in the context of potentially divergent policy scenarios. Specifically, research using the STS/ATS will evaluate natural experiments resulting from differential implementation of systems-level interventions within and between the devolved nations. For example, potential changes in the level and trend in population-level behaviour following policies affecting the availability and price of alcohol or tobacco products in one nation can be compared with others where such policies were not enacted. 

The STS/ATS has demonstrated several strengths during data collection in England to date, and which will now be extended to Scotland and Wales. The surveys have proven to be a valid and valuable tool to examine changes in the prevalence of smoking and harmful drinking at the population level in England (see www.smokinginengland.info) and allowed other important indicators of behaviour such as attempts and motivation and methods to quit/cut down to be assessed regularly ^6^ and in response to key policies and events ^36, 42^. The collection of a large sample of data on a monthly basis has permitted a more sensitive assessment of the effects of these policies than has been possible with annual national surveys. Moreover, all outcomes can be examined according to key socio-demographic characteristics of the population such as socio-economic position, sex, age and region. The combination of smoking and alcohol behaviour measurement also allows comparisons to be made between tobacco and alcohol use ^43, 44^. Finally the data can inform the forecasting of longer-term trends in behaviour and the associated health impacts across GB ^45^.

A limitation of the STS/ATS includes the self-reporting of both smoking and alcohol use. In the context of the COVID-19 pandemic, the temporary replacement of face to face with telephone data collection (and the future return of face to face) may influence the representativeness of the surveys and their comparison with pre-COVID-19 waves. However, the comparability of the two modes of data collection will be assessed using parallel waves of face to face and telephone surveys when possible.

## Data availability

The data referenced by this article are under copyright with the following copyright statement: Copyright: ï¿½ 2021 Kock L et al.

Data associated with the article are available under the terms of the Creative Commons Zero "No rights reserved" data waiver (CC0 1.0 Public domain dedication). 

### Underlying data

No data are associated with this article.

### Extended data

Open Science Framework: Protocol for expansion of an existing national monthly survey of smoking behaviour and alcohol use in England to Scotland and Wales: The Smoking and Alcohol Toolkit Study. https://doi.org/10.17605/OSF.IO/2Z3TE ^21^

This project contains the following extended data:

- A detailed measures table for the STS/ATS (Supplementary Table s1)

Data are available under the terms of the Creative Commons Zero "No rights reserved" data waiver (CC0 1.0 Public domain dedication).
