# Supplementary material for: Protocol for expansion of an existing national monthly survey of smoking behaviour and alcohol use in England to Scotland and Wales: The Smoking and Alcohol Toolkit Study
Source: Wellcome Open Res. 2021 Mar 29;6:67. [Version 1] doi: 10.12688/wellcomeopenres.16700.1 (PMC8370132; doi:10.12688/wellcomeopenres.16700.1)
Supplement: Peer review history

# Peer review: Reviewer response for version 1

**DOI:** 10.21956/wellcomeopenres.18415.r45301

**Published:** 2021-08-16

**Authors:** Chris Bullen

**Affiliations:** 1 National Institute for Health Innovation, University of Auckland, Auckland, New Zealand

The article outlines the study protocol for a series of monthly cross-sectional computer-assisted telephone household surveys on smoking and alcohol use behaviour (and some in England, Scotland and Wales.

 The aims are clear: to provide regular tracking of smoking and alcohol-related behaviours on a monthly basis to inform and evaluate national tobacco and alcohol control policies across devolved nations in Great Britain. There is a strong rationale provided for such analyses.

 Specifically, the surveys will provide a measure of smoking prevalence, ask about smoking and quitting behaviour, the types of support people use to quit and use of e-cigarettes; for alcohol, a measure of hazardous drinking using the AUDIT test will be possible, as well as information on consumption reduction efforts and support for consumption reduction from health professionals. In addition there will be questions about support for policies in play or about to be enacted.

 The survey methods are well-described in sufficient detail to enable others to replicate the approach in other jurisdictions. The survey will be conducted by a market research company. Participants are sampled using a two-stage stratified random location sampling method that has been used in previous waves of the same study but confined to date to England. The sample size is likely to be around 30,000 per year in total.

 More details should be provided about the sample size estimation. Will the smaller samples for Wales and Scotland be sufficient to enable separate country analyses, and inter-country comparisons (which would be informative because of the different policies in each country towards eg e-cigarettes)?

 There is a clear description of the questions but limited reference to validated scales for a number of the questions. There is a lucid account of the analysis of the data including handling of missing data. The authors have a long track record of undertaking such analyses in a rigorous, robust and transparent manner.

Is the study design appropriate for the research question?

Yes

Is the rationale for, and objectives of, the study clearly described?

Yes

Are sufficient details of the methods provided to allow replication by others?

Yes

Are the datasets clearly presented in a useable and accessible format?

Not applicable

Reviewer Expertise:

Epidemiology, public health, tobacco control

I confirm that I have read this submission and believe that I have an appropriate level of expertise to confirm that it is of an acceptable scientific standard.

# Peer review: Reviewer response for version 1

**DOI:** 10.21956/wellcomeopenres.18415.r43321

**Published:** 2021-05-17

**Authors:** Gideon St Helen

**Affiliations:** 1 Clinical Pharmacology Research Program, Division of Cardiology, Department of Medicine, University of California, San Francisco, San Francisco, CA, USA

The protocol to expand an established survey to Scotland and Wales is well-written, the rationale for the protocol/study is clear, and the knowledge to be gained by this expansion is important. For example, the frequent interviews done across the three jurisdictions will allow a comparison of the impact of various policies.

 The protocol does not have much information on questions, if at all, that would address e-cigarette use. Is that left to another study?

 Also, under smoking status, it would be useful to probe on day and time of last use of the products. Further, are cigarettes smoked factory-manufactured or roll-your-own? As more restrictions are placed on smoking, some may move to rolling their own cigarettes. 

 All in all, a well-thought protocol.

Is the study design appropriate for the research question?

Yes

Is the rationale for, and objectives of, the study clearly described?

Yes

Are sufficient details of the methods provided to allow replication by others?

Yes

Are the datasets clearly presented in a useable and accessible format?

Not applicable

Reviewer Expertise:

clinical pharmacology, public health, tobacco control, tobacco regulatory science, toxicology

I confirm that I have read this submission and believe that I have an appropriate level of expertise to confirm that it is of an acceptable scientific standard.
